# Dissecting the Purinergic Signaling Puzzle

**DOI:** 10.3390/ijms22168925

**Published:** 2021-08-19

**Authors:** Raquel Pérez-Sen, Esmerilda G. Delicado

**Affiliations:** Departamento de Bioquímica y Biología Molecular, Facultad de Veterinaria, Instituto Universitario de Investigación en Neuroquímica (IUIN), Instituto de Investigación Sanitaria del Hospital Clínico San Carlos (IdiSSC), Universidad Complutense Madrid, 28040 Madrid, Spain

Purinergic signaling regulates a plethora of physiological processes and is an expanding research field. The main feature of the purinergic network is its complexity, and numerous components integrating into a multistep cascade called a “purinome”. To better understand the physiopathological relevance of nucleotide signaling, it requires to be considered as a whole, having an integrative view of all these elements and their relationships. It includes P1 (adenosine) and P2 (nucleotide) receptors and the enzymatic and transporter activities interconverting nucleotides and nucleosides, connecting the intra- and extracellular spaces to regulate purinergic messenger availability. P1 adenosine receptors are metabotropic receptors belonging to the GPCRs family [1]. They couple differently to G proteins, Gs for the A_2A_ (A_2A_R) and A_2B_ (A_2B_R) receptors, and Gi, for the A_1_ (A_1_R) and A_3_ (A_3_R) receptors. The most widely distributed are the A_1_R and A_2A_R receptors, showing also the highest affinity for the adenosine nucleoside. P2 nucleotide receptors are classified into two groups: metabotropic P2Y receptors (P2YRs) and ionotropic P2X receptors (P2XRs) [2,3]. P2YRs are activated by adenine and/or pyrimidine nucleotides, and couple to different G proteins, and ionotropic P2XRs are ATP-gated cationic channels. Among the plethora of enzymes involved in the nucleotide and adenosine metabolism, ectonucleoside triphosphate diphosphohydrolase/CD39 stands out as a member of NTPDase family, which degrades adenosine 5′-triphosphate (ATP) to the diphosphate ADP, as well as the ectonucleotidase/CD79, which transforms adenosine 5′-monophosphate (AMP) to the nucleoside adenosine. Adenosine can be also metabolized into inosine by the adenosine deaminase activity. Finally, nucleosides are internalized by specific membrane transporters, being the last step of purinergic signaling. Nucleoside transporters include both facilitative and concentrative transporters, which exhibit different substrate and inhibitor selectivity. The aim of this Special Issue was to cover the different components of the purinergic system, regarding how they contribute in a coordinated fashion to shape the final biological response. We consider that the objectives were achieved and greatly thank all the authors. Nowadays researchers receive numerous daily invitations to participate in special issues of different journals and it is difficult to cope with so many invitations.

The issue includes research articles and reviews mainly focused on adenosine and nucleotide receptors in the cardiovascular and nervous systems, the two systems that have been widely approached since the beginning of the purinergic history.

Several contributions point out the dual function of P2X4R in the cardiovascular and nervous systems. Brangança and Correia-de-Sa summarize recent studies concerning the protective role that P2X4R plays in the heart, blood vessels, lung and kidney, which are essential organs for the maintenance of cardiovascular function and its pathophysiological implications. Drug development based on selective activation of the P2X4R could be useful to reduce the burden of cardiovascular diseases [4]. The role of P2X4R in the heart is also confirmed by the study of Woo and Trinh [5]. This work also covers the participation of other ionotropic (P2X7) and metabotropic (P2Y_1_, P2Y_2_ and P2Y_6_) receptors in the cardiac function. Advancing in cardiovascular system, Aslam et al. give an overview of P1 and P2 receptors in endothelial barrier function and illustrate the diverse roles that A2 receptors play on different endothelial beds [6]. Whereas A2R activation results in disruption of endothelial barrier in the blood–brain barrier and coronary microvasculature, it leads to barrier stabilization in lung microvasculature and macrovascular endothelium. The participation of P2X4R, P2X7R and various P2Y receptors in endothelial permeability is also included, indicating that endothelial barrier properties are modulated by multiple purinergic receptors, the final response depending on the local concentrations reached by nucleotides.

Similarly, Montilla et al. summarize the contribution of P2X4R to CNS function as a mediator of synaptic transmission and long-term potentiation, and its pathological implications opening new questions about the use of pharmacological agents that enhance or inhibit P2X4 activity would offer a therapeutic value. The different spatial distribution of P2X2 and P2X4 receptors at the synapsis could represent a new player for fine-tuning of glutamatergic transmission [7]. In this regard, Rodriguez et al. describe an interesting cross-talk between heterologous-expressed P2X4/P2X2 and NMDA receptors in *Xenopus laevis* oocytes. By two-electrode voltage-clamp electrophysiology experiments, authors demonstrate that co-activation of ATP and NMDA receptors induces non-additive responses, which are significantly smaller than predicted and independent of GluN2 subunit composition of NMDARs and extracellular calcium. Using mutagenesis and molecular biology approaches, they conclude that the C-terminal of P2X subunits mediates the interaction between P2X and NMDARs [8].

In line with heteromers, Lillo et al. describe a new heteromer formed by A_2A_ and A_3_ receptors that could operate in the nervous system [9]. The main feature of this heteromeric complex is that A_3_R functionality is negligible, and selective A_2A_R antagonists release the brake on A_3_R activation. This peculiar interaction may be important in drug discovery, as the selective A_2A_R antagonist, istradefylline, has been approved in Japan and USA as an adjuvant therapy in Parkinson’s disease. This study adds a new piece in the complex puzzle of purinergic signaling and suggests that other not yet characterized heteromers could come up.

Another study of the issue related to co-transmission is that of Ziganshin et al., revisiting the effects of ATP and adenosine on quantal and non-quantal acetylcholine release, as well as their postsynaptic actions at the neuromuscular junction [10]. At the presynaptic level, ATP acting on P2Y nucleotide receptors decreases ACh secretion in most studied preparations, whereas adenosine affects secretion either by inhibition via A_1_ receptors or by facilitation through A_2__A_ receptors. At the postsynaptic level, adenosine has no effect at all and the ATP effects are unpredictable. Whereas ATP inhibits postsynaptic transmission at the neuromuscular junction of fast muscles, it displayed a potentiatory effect at the synapses of the slow soleus muscles. ATP actions are more pronounced with hypothermia. Data discussed here suggest that the role of endogenous purines is acting primarily to provide a safety factor that maintains the efficiency of cholinergic neuromuscular transmission, as well as open new prospects for targeted pharmacological regulation of quantal ACh secretion.

Another study covering the implication of purinergic signaling in the nervous system is that of Sánchez-Melgar et al., which analyzes the changes in the adenosine metabolism occurring in the cerebral cortex from several murine models of aging: senescence-accelerated mouse-resistant 1 (SAMR1, normal senescence), senescence-accelerated mouse-prone 8 (SAMP8, a model of Alzheimer’s disease), and the wild-type C57 BL/6 J mice strains [11]. The major changes are observed in the cerebral cortex of SAMP8 mice at the age of six months, which show a reduction of 5′-nucleotidase and AMPK activities and low levels of inosine, hypoxanthine and guanosine compared to those observed in SAMP1 and C57 BL/6 J mice. In addition, glutamate and excitatory amino acid transporter 2 (EAAT2) levels are also diminished, which is in line with cognitive decline observed in the SAMP8 strain. As SAMP8 is an Alzheimer’s disease model, data suggest that adenosine metabolism activity is involved in this neurodegenerative disease.

Other receptors with therapeutic potential to maintain nervous system functionality are P2Y_12_R and GPR17. The study reported by Lowery et al. investigates the involvement of P2Y_12_R in the development of murine lateral geniculate nucleus and primary somatosensorial cortex and behavior performance in adulthood. They prove that P2Y_12_R is required neither for developmental retinogeniculate reorganization in the lateral geniculate nucleus nor for microglial infiltration into thalamocortical axon clusters of S1 barrel. In adult mice; however, P2Y_12_R becomes essential for synaptic plasticity, as P2Y_12_^−/−^ mice exhibit alterations in learning and social behavior [12]. It is important to confirm the role of P2Y_12_R in neuronal circuitry development and synaptic plasticity to design new therapeutic approaches for cognitive dysfunction, keeping in mind that P2Y_12_ receptor antagonists are administrated world-wide as a platelet drug for the treatment of coronary thrombosis. The contribution of Gasecka et al. deals with another interesting aspect to consider for the diagnosis and treatment of coronary thrombosis, the platelet extracellular vesicles (PEVs) released in basal conditions and after platelet activation [13]. Authors summarize and discuss the contribution of P2Y_1_ and P2Y_12_ receptors to these processes and the underlying molecular mechanisms. Bonfanti et al. demonstrate the implication of the GPR17 receptor in oligodendrocyte dysfunction in the mouse disease model of amyotrophic lateral sclerosis (ALS) [14]. They show that oligodendroglial GPR17R expression is upregulated in the spinal cord of SOD1G93 mice. Using primary cultures of oligodendrocyte precursors (OPCs) from the spinal cord of P7 SOD1G93 and WT mice, they clearly prove that the GPR17R upregulation disturbs differentiation and maturation of OPCs from P7 SOD1G93 mice, as the antagonist montelukast restores their differentiation capabilities. Data noticeably point to the GPR17R as a potential pharmacological tool to develop novel therapeutic approaches to counteract oligodendrocytes dysfunction in ALS, and to retard motoneuron degeneration.

The issue also includes a stimulating study revealing that atomic force microscopy (AFM) can be another tool to evaluate functional native nucleotide receptors in living cells. AFM in the so-called force spectroscopy mode allows simultaneous determinations of variations in parameters such as Young´s modulus, maximum adhesion force and rupture event formation, which reflect changes in both the stiffness and adhesiveness of the plasma membrane. Gil-Redondo et al. demonstrate that stimulation of P2Y_2_R in endothelial (HUVEC) cells and astrocytes doubles the Young´s modulus and reduces the adhesion force and the number of rupture events. In astrocytes, P2Y_1_/P2Y_13_ receptor activation also modify AFM parameters. The results indicate that nucleotides alter mechanical properties of the plasma membrane in living cells modifying cytoskeleton rearrangements, focal adhesion kinase (FAK) being one of the targeted proteins [15].

Completing the contributions about P2Y receptors, the interesting report of De la Rosa et al. adds new data on the relevant role that nucleotides play in macrophage function. They show that P2Y_2_ receptor activation increases LPS-primed IL-1β production [16]. The enhancement is dependent on c-Jun N-terminal kinase activation and independent of NPRP3 inflammasome activation. The results indicate that, in addition to P2X7R, P2Y_2_R could be another relevant target to treat inflammation.

Additionality, other contributions are related to enzymatic activities responsible for nucleotide and adenosine metabolism, like adenosine deaminase and ectonucleotide triphosphate diphosphohydrolase-2 and their implications in drug therapies. In this regard, Cones-Buendía et al. analyze the effect on two formulations of tenofovir, a nucleoside reverse transcriptase inhibitor analog (TDF, Tenofovir disoproxil fumarato and TAF, Tenofovir alafenamida) and Triumeq (a combination of reverse transcriptase and integrase inhibitors, abacavir/dolutegravir/lamivudine) on adenosine deaminase and cytokine levels in treatment-naïve HIV patients. They observe that ADA concentrations increase (over 15 U/L) in treatment-naïve HIV patients and decrease after treatment with TAF and Triumeq, though this does not occur in TDF-treated patients. However, all groups exhibited a pro-inflammatory systemic profile after 12 months of treatment. They conclude that antiretroviral therapy in HIV patients may modulate ADA levels and differentially affect inflammatory status [17].

The article of Feldbrügge et al. reveals new and compelling data about the neuroprotective role of ectoenzymes degrading nucleotides in liver injury using an experimental model of acetaminophen-induced hepatotoxicity. They show that ectonucleotidase triphosphate diphosphohydrolase-2 (NTPDase2) expression is upregulated after acetaminophen-induced acute live injury. *Entpd2* null mice display more hepatic necrosis, higher levels of serum alanine aminotransferase and hepatic IL-6 and PDGF-B than observed in wild-type animals. The increase of IL6 and PDGF-B observed in *Entpd2* null mice would stimulate liver regeneration, which indicates the mitigating effect of NTPDase 2 in the initial necrotizing phase of liver injury [18].

Another study of the issue reveals the implication of purinergic signaling in the clinics of human organ transplantation. Guillén-Gómez et al. evaluate differences in purinergic gene expression (nucleoside concentrative transporter, adenosine receptors, enzymes responsible for nucleotide metabolism and Panexin 1) in pre-implantation human tissue samples obtained from kidney biopsies of deceased (DD) and living (LD) donors. DD kidney grafts show high mRNA levels of Panx1 and A_2A_R and low levels of CD73, EPP3 and CNT2, in comparison to those observed in LD kidney grafts [19]. They conclude that DD kidney grafts display more inflammation and produce high level of TNF-α. TNF-α via A2AR induces CD163 and TFG-β1 expression and initiates anti-inflammatory processes and ultimately fibrosis, which may contribute to graft dysfunction and prognostic differences between DD and LD transplants.

In the context of tumor progression, Novak et al. review the role of purinergic signaling in pancreatic ductal adenocarcinoma (PDAC). Data reported in the literature reveal that PX7R, P2Y_2_R, A_2B_R and the ectoenzymes CD39 and CD73 are upregulated in the different cellular types existing in the tumor environment, pancreatic ductal adenocarcinoma cells, pancreatic stellate cells and immune cells, and act in a coordinated way to support oncogenesis, fibrosis and inflammation. There are some drugs or antibodies targeting one or more components of purinergic signaling engaged in clinical trials for PDAC treatment [20].

In conclusion, as mentioned previously, we believe that the studies presented here cover multiple aspects of the ongoing research in the purinergic field and show the difficult task of approaching the complexity of this ubiquitous system. Finally, we thank again all the colleagues and people that have contributed with their work to this Special Issue, and we hope that its content is of interest for additional researchers aiming to explore this thrilling field.

## References

[1] Borea P.A., Gessi S., Merighi S., Vincenzi F., Varani K. (2018). Pharmacology of Adenosine Receptors: The State of the Art. Physiol. Rev..

[2] Jacobson K.A., Delicado E.G., Gachet C., Kennedy C., von Kugelgen I., Li B., Miras-Portugal M.T., Novak I., Schoneberg T., Perez-Sen R. (2020). Update of P2Y receptor pharmacology: IUPHAR Review 27. Br. J. Pharmacol..

[3] Illes P., Muller C.E., Jacobson K.A., Grutter T., Nicke A., Fountain S.J., Kennedy C., Schmalzing G., Jarvis M.F., Stojilkovic S.S. (2021). Update of P2X receptor properties and their pharmacology: IUPHAR Review 30. Br. J. Pharmacol..

[4] Braganca B., Correia-de-Sa P. (2020). Resolving the Ionotropic P2X4 Receptor Mystery Points Towards a New Therapeutic Target for Cardiovascular Diseases. Int. J. Mol. Sci..

[5] Woo S.H., Trinh T.N. (2020). P2 Receptors in Cardiac Myocyte Pathophysiology and Mechanotransduction. Int. J. Mol. Sci..

[6] Aslam M., Gunduz D., Troidl C., Heger J., Hamm C.W., Schulz R. (2021). Purinergic Regulation of Endothelial Barrier Function. Int. J. Mol. Sci..

[7] Montilla A., Mata G.P., Matute C., Domercq M. (2020). Contribution of P2X4 Receptors to CNS Function and Pathophysiology. Int. J. Mol. Sci..

[8] Rodriguez L., Yi C., Chu C., Duriez Q., Watanabe S., Ryu M., Reyes B., Asatryan L., Boue-Grabot E., Davies D. (2020). Cross-Talk between P2X and NMDA Receptors. Int. J. Mol. Sci..

[9] Lillo A., Martinez-Pinilla E., Reyes-Resina I., Navarro G., Franco R. (2020). Adenosine A2A and A3 Receptors Are Able to Interact with Each Other. A Further Piece in the Puzzle of Adenosine Receptor-Mediated Signaling. Int. J. Mol. Sci..

[10] Ziganshin A.U., Khairullin A.E., Hoyle C.H.V., Grishin S.N. (2020). Modulatory Roles of ATP and Adenosine in Cholinergic Neuromuscular Transmission. Int. J. Mol. Sci..

[11] Sanchez-Melgar A., Albasanz J.L., Pallas M., Martin M. (2020). Adenosine Metabolism in the Cerebral Cortex from Several Mice Models during Aging. Int. J. Mol. Sci..

[12] Lowery R.L., Mendes M.S., Sanders B.T., Murphy A.J., Whitelaw B.S., Lamantia C.E., Majewska A.K. (2021). Loss of P2Y12 Has Behavioral Effects in the Adult Mouse. Int. J. Mol. Sci..

[13] Gasecka A., Rogula S., Eyileten C., Postula M., Jaguszewski M.J., Kochman J., Mazurek T., Nieuwland R., Filipiak K.J. (2020). Role of P2Y Receptors in Platelet Extracellular Vesicle Release. Int. J. Mol. Sci..

[14] Bonfanti E., Bonifacino T., Raffaele S., Milanese M., Morgante E., Bonanno G., Abbracchio M.P., Fumagalli M. (2020). Abnormal Upregulation of GPR17 Receptor Contributes to Oligodendrocyte Dysfunction in SOD1 G93A Mice. Int. J. Mol. Sci..

[15] Gil-Redondo J.C., Iturri J., Ortega F., Perez-Sen R., Weber A., Miras-Portugal M.T., Toca-Herrera J.L., Delicado E.G. (2021). Nucleotides-Induced Changes in the Mechanical Properties of Living Endothelial Cells and Astrocytes, Analyzed by Atomic Force Microscopy. Int. J. Mol. Sci..

[16] de la Rosa G., Gomez A.I., Banos M.C., Pelegrin P. (2020). Signaling Through Purinergic Receptor P2Y2 Enhances Macrophage IL-1beta Production. Int. J. Mol. Sci..

[17] Conesa-Buendia F.M., Llamas-Granda P., Atencio P., Cabello A., Gorgolas M., Largo R., Herrero-Beaumont G., Mediero A. (2020). Adenosine Deaminase as a Biomarker of Tenofovir Mediated Inflammation in Naive HIV Patients. Int. J. Mol. Sci..

[18] Feldbrugge L., Splith K., Kammerer I., Richter S., Riddermann A., Ortiz Galindo S.A., Krenzien F., Muller T., Csizmadia E., Pratschke J. (2020). Ecto-Nucleotide Triphosphate Diphosphohydrolase-2 (NTPDase2) Deletion Increases Acetaminophen-Induced Hepatotoxicity. Int. J. Mol. Sci..

[19] Guillen-Gomez E., Silva I., Serra N., Caballero F., Leal J., Breda A., San Martin R., Pastor-Anglada M., Ballarin J.A., Guirado L. (2020). From Inflammation to the Onset of Fibrosis through A2A Receptors in Kidneys from Deceased Donors. Int. J. Mol. Sci..

[20] Novak I., Yu H., Magni L., Deshar G. (2020). Purinergic Signaling in Pancreas-From Physiology to Therapeutic Strategies in Pancreatic Cancer. Int. J. Mol. Sci..

